# LINC00460 Is a Dual Biomarker That Acts as a Predictor for Increased Prognosis in Basal-Like Breast Cancer and Potentially Regulates Immunogenic and Differentiation-Related Genes

**DOI:** 10.3389/fonc.2021.628027

**Published:** 2021-04-12

**Authors:** Mireya Cisneros-Villanueva, Lizbett Hidalgo-Pérez, Alberto Cedro-Tanda, Mónica Peña-Luna, Marco Antonio Mancera-Rodríguez, Eduardo Hurtado-Cordova, Irene Rivera-Salgado, Alejandro Martínez-Aguirre, Silvia Jiménez-Morales, Luis Alberto Alfaro-Ruiz, Rocío Arellano-Llamas, Alberto Tenorio-Torres, Carlos Domínguez-Reyes, Felipe Villegas-Carlos, Magdalena Ríos-Romero, Alfredo Hidalgo-Miranda

**Affiliations:** ^1^ Laboratorio de Genómica del Cáncer, Instituto Nacional de Medicina Genómica (INMEGEN), Ciudad de México, México; ^2^ Laboratorio de Epigenética del Cáncer, Facultad de Ciencias Químico Biológicas, Universidad Autónoma de Guerrero, Chilpancingo de los Bravo, Mexico; ^3^ Programa de Doctorado en Ciencias Biomédicas, Facultad de Medicina, Universidad Nacional Autónoma de México (UNAM), Ciudad de México, Mexico; ^4^ Departamento de Anatomía Patológica, Hospital Central Sur de Alta Especialidad, Petróleos Mexicanos, Ciudad de México, México; ^5^ Instituto de Enfermedades de la Mama, FUCAM, Ciudad de México, Mexico; ^6^ Posgrado en Ciencias Biológicas, Unidad de Posgrado, Universidad Nacional Autónoma de México (UNAM), Ciudad de México, México

**Keywords:** LINC00460, breast cancer, basal-like, biomarker, increased prognosis, mir-103a, sponge, WNT7A

## Abstract

Breast cancer (BRCA) is a serious public health problem, as it is the most frequent malignant tumor in women worldwide. BRCA is a molecularly heterogeneous disease, particularly at gene expression (mRNAs) level. Recent evidence shows that coding RNAs represent only 34% of the total transcriptome in a human cell. The rest of the 66% of RNAs are non−coding, so we might be missing relevant biological, clinical or regulatory information. In this report, we identified two novel tumor types from TCGA with LINC00460 deregulation. We used survival analysis to demonstrate that LINC00460 expression is a marker for poor overall (OS), relapse-free (RFS) and distant metastasis-free survival (DMFS) in basal-like BRCA patients. LINC00460 expression is a potential marker for aggressive phenotypes in distinct tumors, including HPV-negative HNSC, stage IV KIRC, locally advanced lung cancer and basal-like BRCA. We show that the LINC00460 prognostic expression effect is tissue-specific, since its upregulation can predict poor OS in some tumors, but also predicts an improved clinical course in BRCA patients. We found that the LINC00460 expression is significantly enriched in the Basal-like 2 (BL2) TNBC subtype and potentially regulates the WNT differentiation pathway. LINC00460 can also modulate a plethora of immunogenic related genes in BRCA, such as *SFRP5, FOSL1, IFNK, CSF2, DUSP7* and *IL1A* and interacts with miR-103-a-1, *in-silico*, which, in turn, can no longer target WNT7A. Finally, LINC00460:WNT7A ratio constitutes a composite marker for decreased OS and DMFS in Basal-like BRCA, and can predict anthracycline therapy response in ER-BRCA patients. This evidence confirms that LINC00460 is a master regulator in BRCA molecular circuits and influences clinical outcome.

## Introduction

Breast cancer (BRCA) is a major public health problem, as it is the most frequent malignant tumor in women worldwide. According to GLOBOCAN (2018), at least 2.088 million new cases and a total of 626,679 deaths were reported globally (1). Breast cancer is a phenotypically heterogeneous disease, with well-defined histological types. In 2000, Perou et al. (2), suggested that this physical heterogeneity is also reflected on molecular level, particularly on transcriptome, where same histological types can display a variety of differentially expressed genes. Four BRCA subgroups are differentiated by the expression of 50 genes (the PAM50 signature): Luminal A, Luminal B, HER2-enriched and Basal-like tumors. This molecular classification has been used to discern between aggressive and non-aggressive tumors, evaluate metastatic potential, establish clinical prognosis and estimate survival, among other relevant cancer-related features. Furthermore, patients´ therapy response-associated expression profiles can be subjected to the same classification, allowing us to predict a type of therapy a patient can benefit from, e.g. chemotherapy or anti-hormonal therapy (3). All these clinical advances are only focused on coding RNAs profiles; however, more recent reports have shown that coding (messenger) RNAs represent only 2% of the total transcriptome in a human cell (4). The remaining 98% of transcriptome are non-coding RNAs, that might nevertheless carry relevant biological and clinical information.

Long non-coding RNAs (lncRNAs) are non-translated transcripts with a length of 200 nucleotides or superior. In recent years, several papers have focused on their role in different types of cancer showing their contribution to critical biological processes including carcinogenesis, apoptosis, differentiation, proliferation, invasion and metastasis among others (5–8). LncRNAs display multiple regulatory functions, as they can act as modulators of transcription and chromatin remodeling (9, 10), as splicing factors (11, 12), as regulators of mRNA decay (13), mRNA stability (14), as protein decoys (15) and microRNA (miRNA) sponges (16, 17). In this sponging effect, a lncRNA competes with a miRNA to release the inhibition of other genes (18, 19). Several reports have shown that sponge lncRNAs play a pivotal role in various cancer types (20–24), including BRCA (25, 26) and abnormal expression of lncRNAs can significantly contribute to BRCA initiation and progression (25, 27, 28).

The long intergenic non-coding RNA 460 (LINC00460) is a human lncRNA gene, transcribed from chromosome 13q33.2 and measuring 913 bp (29). The LINC00460 is constituted by three exons and it has been shown that it lacks coding capability (30, 31). According to the ENSEMBL database, the human LINC00460 gene has seven splice variant transcripts reported to date (32). It has been observed that LINC00460 functions as an oncogene, acting as a miRNA sponge (17, 33–43). In these models, LINC00460 sponging activity and its association with cancer-related processes such as increase in proliferation, epithelial to mesenchymal transition (EMT), migration, invasion and metastasis (44–46) is well described. For example, LINC00460 promotes hepatocellular carcinoma progression by sponging miR-342-3p and hence increasing AGR2 expression level (47). Similar LINC00460 sponging and cancer-related mechanisms across distinct tumors are described in several reports (38–43, 48).

The LINC00460 role in clinical cancer features has also been studied. LINC00460 over-expression is strongly associated with poor survival rates in different tumors, such as lung, ovary, larynx, nasopharynx, head and neck, meningioma, kidney, thyroid, colorectal, glioma, osteosarcoma, bladder and cervix (31, 35, 48–56). Thus, LINC00460 is a well-defined sponge lncRNA with significant prognostic potential in several tumors, however its clinical and biological relevance in BRCA is poorly understood (33), and thorough studies are needed.

In this study, we aimed to elucidate the potential role of LINC00460 in BRCA. For this purpose, we performed a series of analysis to infer its biological relevance and verify the underlying role of LINC00460 in BRCA. We identified two novel (not previously reported) tumor types from the TCGA cohort with LINC00460 deregulation. In addition, we used a multivariate Cox regression analysis to demonstrate that LINC00460 expression is related to poor prognosis in three different tumors. We show here that LINC00460 upregulation is significantly associated with improved survival in BRCA in three independent cohorts. This increased survival effect is replicated in Basal-like BRCA and furthermore, we demonstrate that LINC00460 is significantly enriched in the Basal-like 2 (BL2) Triple Negative Breast Cancer (TNBC) subtype. Using differential expression and correlation analysis, we show that LINC00460 overexpression impairs several breast cancer-related pathways: WNT signaling pathway, cytokine inflammatory response and DNA damage response. Among the most relevant potential LINC00460 gene targets, we found *WNT7A, SFRP5, FOSL1* and *IFNK* to be deregulated in the LINC00460-high condition. Using *in-silico* prediction analysis, we show that LINC00460 could interact with miR-103-a, which in turn, potentially regulates *WNT7A.* Finally, we demonstrate that LINC00460: WNT7A ratio is a composite marker for increased OS and DMFS in Basal-like BRCA, and their combined expression can predict anthracycline therapy response in ER-BRCA patients, which further pinpoints the biological and clinical role of these transcripts in TNBC.

## Materials and Methods

### The Cancer Genome Atlas (TCGA) Datasets

LINC00460 expression levels were screened in 31 TCGA tumor datasets and their corresponding GTEx normal tissues using the Gene Expression Profiling Interactive Analysis 2 (GEPIA2) platform (http://gepia2.cancer-pku.cn/#index) (57). The 31 tumors included in the analysis are summarized in **Supplementary Table 1** and enlisted as follows: Acute myeloid leukemia (LAML); Adrenocortical carcinoma (ACC); Bladder urothelial carcinoma (BLCA); Brain lower grade glioma (LGG); Breast invasive carcinoma (BRCA); Cervical squamous cell carcinoma and endocervical adenocarcinoma (CESC); Cholangiocarcinoma (CHOL); Colon adenocarcinoma (COAD); Esophageal carcinoma (ESCA); Glioblastoma multiforme (GBM); Head and neck squamous cell carcinoma (HNSC); Kidney chromophobe (KICH); Kidney renal clear cell carcinoma (KIRC); Kidney renal papillary cell carcinoma (KIRP); Liver hepatocellular carcinoma (LIHC); Lung adenocarcinoma (LUAD); Lung squamous cell carcinoma (LUSC); Lymphoid neoplasm diffuse large B-cell lymphoma (DLBC); Ovarian serous cystadenocarcinoma (OV); Pancreatic adenocarcinoma (PAAD); Pheochromocytoma and Paraganglioma (PCPG); Prostate adenocarcinoma (PRAD); Rectum adenocarcinoma (READ); Sarcoma (SARC); Skin cutaneous melanoma (SKCM); Stomach adenocarcinoma (STAD); Testicular germ cell tumors (TGCT); Thymoma (THYM); Thyroid carcinoma (THCA); Uterine carcinosarcoma (UCS); Uterine corpus endometrial carcinoma (UCEC). Mesothelioma (MESO) and Uveal melanoma (UVM) were removed from the analysis, since the correspondent normal tissues were not available in the GEPIA2 platform.

Additional analysis with TCGA breast cancer, normal tissues and HNSC HPV status, was performed using data retrieved from the TANRIC platform (https://ibl.mdanderson.org/tanric/_design/basic/main.html) (58). Violin plots, box plots and notch plots were constructed using the ggplot2 R package. Validation of LINC00460 over-expression in BRCA versus normal tissues was performed using data retrieved from the GSE29431 dataset (https://www.ncbi.nlm.nih.gov/geo/) and analyzed using the lnCAR platform (https://lncar.renlab.org/) (59).

LUAD, LUSC and KIRC tumor stage plots were generated in the GEPIA2 platform. LINC00460 expression levels were considered significantly correlated with tumors when log2 fold change (Log2FC)>1 and p-value<0.01.

### Breast Cancer Samples Differential Expression Analysis

Breast cancer RNA seq counts were obtained from The Cancer Genome Atlas (TCGA) data portal (https://portal.gdc.cancer.gov). After dataset preparation, we identified the LINC00460 ID (ENSG00000233532), which is located in chromosome 13: 106,376,563-106,378,217. We then downloaded the LINC00460 expression counts and performed filtering of transcripts with 10 counts or less. In order to generate the high and low LINC00460 expression groups, we calculated two percentiles from the count expression data. The first percentile (25) contains the lowest LINC00460 expression counts, and the upper percentile (60), contains the highest expression levels for this transcript. We then performed differential expression analysis with the DESeq2 tool from the Gene Pattern platform, using the default parameters (http://software.broadinstitute.org/cancer/software/genepattern/) (61).

Genes were considered differentially expressed when Log2FC was >1.5 and -<1.5 and p- adjusted value <0.05. Volcano plots were generated with the ggplot2 and ggrepel R packages.

### Breast Cancer Patients and Biological Samples

A total of 74 biological samples (frozen tissues) were collected from breast cancer patients attending Fundación de Cáncer de Mama A.C. (FUCAM) in Mexico City, Mexico. Formalin fixed paraffin embedded (FFPE) breast cancer samples (n=19) were collected from the Hospital Central Sur de Alta Especialidad, Petróleos Mexicanos (PEMEX). None of the patients received neoadjuvant therapy. All patients signed a written informed consent. The studies involving human participants were reviewed and approved by the Research Ethics Committee (INMEGEN) and the FUCAM Ethics Committee (Registration number: CE2009/11).

Biological samples were bisected; one portion was fixed in formaldehyde (10%), paraffin embedded (FFPE) (Paraplast Plus^®^; Sigma Aldrich ^®^, St Louis, Missouri, USA) and then submitted to Hematoxylin and Eosin (H&E) staining for histopathological examination by a certified pathologist. Tumor stage was assessed, according to international standards. The second portion of the sample was used for RNA extraction and functional downstream analysis. All tissues were liquid nitrogen-frozen and stored at -80°C. Samples with more than 80% tumor cells were included in the analysis, otherwise were discarded.

An expert pathologist performed the standardized evaluation of stromal tumor-infiltrating lymphocytes (TILs) based on H&E -stained slides of breast tumoral tissue. Briefly, TILs assessment was performed as follows: a) only TILs within the borders of the invasive tumors were evaluated; b) the invasive edge was included in the evaluation; c) only mononuclear infiltrates were included; d) immune infiltrates in adjacent normal tissue or areas of central necrosis or fibrosis were not included; e) the average TILs of the stromal area were reported. For the purposes of this research, the cut-off points of TILs were defined in less than 50% and more than 50% in the stromal area.

In all cases, demographic (age, sex), clinical (date of diagnosis, therapy received), pathologic (stage, grade, histological type) and prognostic data (recurrence, progression and overall survival) were available and correlated with LINC00460 expression status.

### RNA Extraction

Total RNA was extracted using the commercial kit AllPrep^®^ DNA/RNA FFPE (Qiagen^®^ Inc, Valencia, CA) following manufacturer’s instructions. Briefly, the tissues were deparaffinized, disrupted and lysed. RNA was then precipitated, washed, purified and suspended in RNAse free water. RNA concentration was evaluated by spectrophotometry (NanoDrop Technologies, Wilmington, Delaware, USA). RNA integrity was analyzed using the BioAnalyzer 2100 (Agilent Technologies, Palo Alto, CA, USA), only high-quality samples were used. Samples were stored at -80°C until processing.

### Microarray Re-Analysis

Using the BRCA cohort previously reported by our group in (62) (n = 74; Luminal A = 24, Luminal B = 23, HER2 = 14, Basal = 13) in which samples were analyzed using Human Transcriptome Array 2.0 (Affymetrix, Inc, Santa Clara, CA), we were able to identify LINC00460 expression levels across BRCA subtypes. We used the Robust Multi-chip Analysis (RMA) algorithm to minimize the effect of probe-specific affinity differences and to normalize samples (63). Log 2 relative fluorescent signal intensities were computed using the Transcriptome Analysis Console (Affymetrix, Inc, Santa Clara, CA).

### Quantitative Reverse Transcription Polymerase Chain Reaction (qRT-PCR)

cDNA was synthetized using SuperScript III RT-PCR (Invitrogen, ThermoFisher™ Scientific, Waltham, Massachusetts, USA) and High-Capacity cDNA Reverse Transcription Kit (Applied Biosystems™, Foster City, California, USA), following the manufacturer’s instructions. Briefly, 100 ng of total RNA from cell lines or breast cancer samples were used to synthesize cDNA in a final reaction volume of 20μL. The PCR reaction contained 1μL of cDNA, 5 μL 2X TaqMan Universal Master Mix (Applied Biosystems, ThermoFisher™ Scientific, Waltham, Massachusetts, USA), 0.5 μL TaqMan probes (custom-made for LINC00460) and 3.5 μL of nuclease-free water. Both primers and the reporter were designed to target LINC00460, exon 2, transcript variant 1 (NCBI Reference Sequence: NR_034119.2). Forward primer: CCTGGATGAACCACCATTGC; reverse primer: ATGAGAACGAAGGTTACGACCATT; reporter: ATGTTGCAGCTTTCCCA). GAPDH (Hs99999905) and SCARNA5 (Hs03391742_cn) transcripts were used as endogenous controls.

### Pathway Enrichment Analysis

Pathway enrichment analysis was performed using Ingenuity Pathway Analysis^®^ (IPA) software. Z-scores and p-values were also computed using this platform. Only differential statistically significant genes were included in this analysis (see criteria above).

Overrepresentation Enrichment Analysis (ORA) was performed with the web-based Gene SeT Analysis Toolkit (WebGestalt) platform (www.webgestalt.org) (64). LINC00460 significantly correlated genes were included in this analysis.

### BRCA and HNSC Datasets for Validation

Survival analyses from independent BRCA Gene Expression Omnibus (GEO) cohorts were performed using the Kaplan-Meier plotter (KM plotter) site (http://kmplot.com/analysis/) (65). These GEO datasets contain gene expression data from 6,234 BRCA samples analyzed with multiple microarray platforms, including Human Genome U133 and Human Genome U133 Plus 2.0 Arrays (Affymetrix). We used GEO-derived cohorts, the GSE16446 and GSE21653 datasets, or a combination of several datasets. The GSE16446 dataset was selected, since it contains 120 microarray experiments from primary ER-negative breast tumors of anthracycline-treated patients (66). The GSE21653 dataset contains data of 266 invasive breast adenocarcinomas, with all BRCA subtypes included (67, 68). We used the Affymetrix probe ID 1558930_at, that targets LINC00460, and the Affymetrix probe ID 210248_at, which targets WNT7A. In addition, we performed survival analysis of hsa-miR-103a expression levels, using the mirPower tool (69), included in the KM plotter website.

LINC00460:WNT7A ratio for TNBC survival analyses was also performed in the KM plotter tool. LINC00460 and WNT7A signature expression validation as anthracycline predictive markers were analyzed with the ROC Plotter platform (http://www.rocplot.org/site/index) (70).

For HNSC validation, we downloaded the GEO dataset GSE3292. This cohort has 36 freshly frozen HNSC samples, stratified by HPV status (negative or positive) and analyzed by gene expression microarrays (Affymetrix Human Genome U133 Plus 2.0 Array).

For further TNBC validation, we downloaded the GEO dataset GSE76250, which includes 165 samples. We then analyzed the expression profile of these samples, and classified them with the TNBCtype tool to obtain the Lehman subtypes (http://cbc.mc.vanderbilt.edu/tnbc) (71). We then re-classified these samples into the TNBCtype4 re-assigning IM and MSL subtypes to the second highest correlated centroid, as described in (72). For LINC00460 expression in TNBC cell lines validation, we screened the Cancer Cell Line Encyclopedia (CCLE) online tool (https://portals.broadinstitute.org/ccle) (73).

The validation datasets analyzed for this study can be found in the Gene Expression Omnibus (GEO) web site (https://www.ncbi.nlm.nih.gov/geo/).

### miRNA Interaction Prediction Analysis

We used the miRcode prediction tool (http://www.mircode.org/) (74) in order to identify potential LINC00460 miRNA targets. We further corroborated these potential interactions using the lncTAR tool (http://www.cuilab.cn/lnctar) (75). Interaction prediction was considered valid when normalized delta G (ndG) values reached the -0.1 cutoff.

We then performed a third *in-silico* prediction analysis, using the miRPathDB tool (https://mpd.bioinf.uni-sb.de/) (60). We searched the top 15 correlated mRNAs with LINC00460 expression levels (see **Table 4**) and downloaded the miRNAs interaction list. We then compared these new miRNAs list with the previous one described above, and identified potential miRNA-mRNA-linc00460 interactions.

### Statistical Analysis

It has been previously shown that cancer gene expression profiles are not normally-distributed, either on the complete-experiment or on the individual-gene level (76). Thus, LINC00460 expression distributions from the TCGA and validation BRCA, KIRC, LUAD and HSNC datasets were first tested for normality distribution using the Kolmogorov-Smirnov and the Shapiro-Wilk tests. After results computation, we selected comparison statistical tests accordingly.

Overall Survival (OS) using all patients from CESC, GBM, HNSC, LGG, LUAD, PAAD and SARC and the Relapse Free Survival (RFS) analysis using all patients from KIRC, LUAD, READ and SARC was performed in the GEPIA2 platform. Distant Metastasis Free Survival (DMFS) was computed using the KM plotter tool described above.

OS of the BRCA TCGA patients and our independent cohort was analyzed with the Kaplan-Meier model and the multivariable Cox regression model. Relative Risk was also calculated. This analysis was performed with the PASW statistics software (SPSS, IBM^®^, Quarry Bay, Hong Kong). Chi-square tests were calculated in order to correlate clinical variables status with LINC00460 expression level (high or low expression).

For all statistical tests, the level of significance was <0.05.

## Results

### LINC00460 Expression Is Deregulated in Multiple Tumors

**Figure 1:**
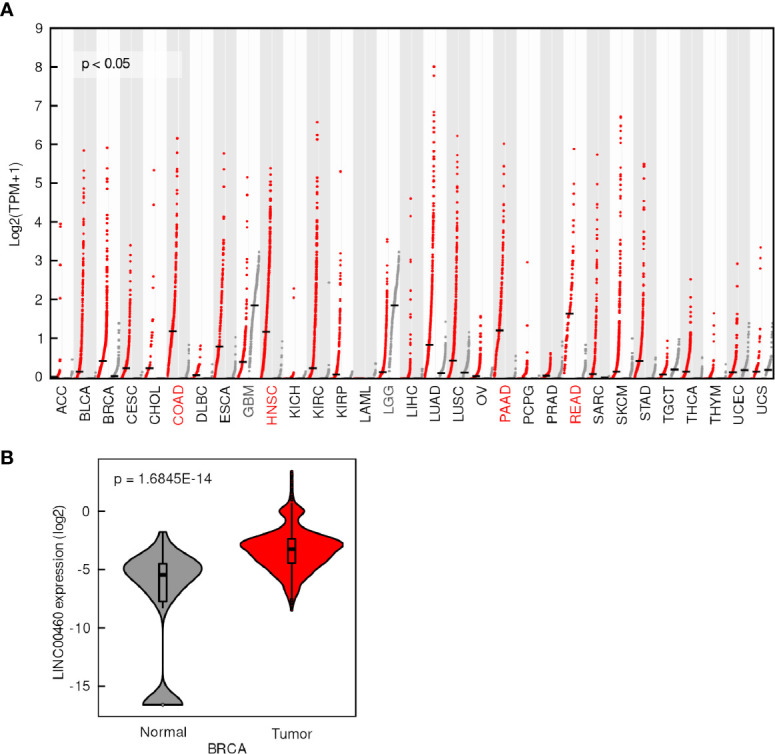
LINC00460 is aberrantly expressed in different tumors. **(A)** LINC00460 expression levels in 33 tumors (red dots) and its correspondent normal tissues (grey dots). Tumors with overexpression and down expression of LINC00460 have red and grey color abbreviations, respectively. Data was obtained from GEPIA2 and modified. **(B)** LINC00460 is over-expressed in breast cancer compared with normal tissues. Data was retrieved from TANRIC and then re-plotted.

LINC00460 expression was evaluated in 31 tumor types and normal tissues included in TCGA database. We found that LINC00460 is overexpressed in five different epithelial cancers, namely: Breast cancer (BRCA) (**Figure 1B**); Colon adenocarcinoma (COAD); Head and Neck Squamous cell carcinoma (HNSC), Pancreatic adenocarcinoma (PAAD) and Rectum adenocarcinoma (READ) (Log2FC>1; p<0.05) (**Figure 1A**). Interestingly, we have also detected two central nervous system cancers with LINC00460 low expression, comparing with its normal tissue counterparts: Glioblastoma Multiforme (GBM) and Low-Grade Glioma (LGG) (Log2FC<1; p<0.01) (**Supplementary Figure S1**). These observations potentially suggest a different role for LINC00460 in central nervous system tumors, which can further be analyzed in future studies.

Among the seven LINC00460-deregulated tumors from the TCGA cohort, we identified two novel (not previously reported) tumors, namely LGG and GBM (**Supplementary Figure S1**). We have also confirmed previous observations regarding LINC00460 overexpression in BRCA (33), comparing with normal tissue, using two independent cohorts, namely TCGA (Tanric data, T test; p=1.6845E-14) and the GEO cohort GSE29431 (**Figure 1** and **Supplementary Figure S2**). These observations suggest a ubiquitous role for LINC00460 in cancer biology.

### LINC00460 Expression Is Associated With Advanced Clinical Stages and Aggressive Phenotypes in Different Cancers

**Figure 2:**
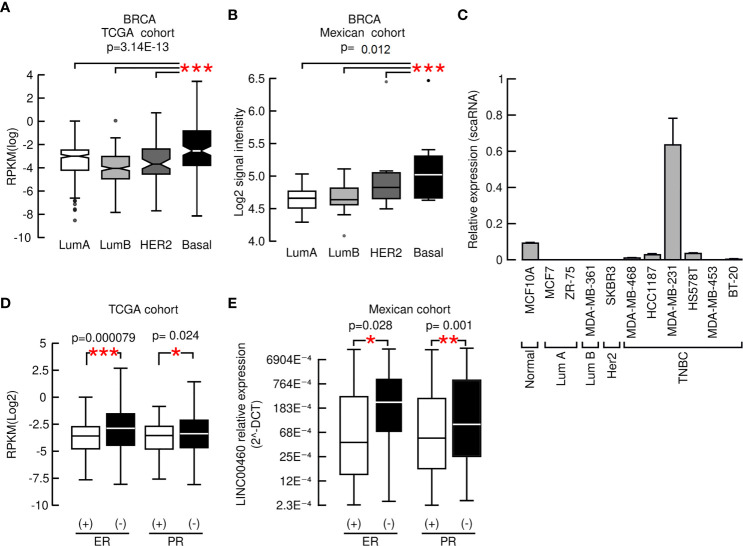
LINC00460 high expression is associated with aggressive phenotypes in BRCA. **(A)** LINC00460 expression is significantly related with Basal-like BRCA in the TCGA cohort, **(B)** in a Mexican independent cohort and **(C)** enriched in TNBC cell lines, **(D)** LINC00460 is significantly enriched in ER- and PR- BRCA in the TCGA and **(E)** an independent Mexican BRCA cohort. *p<0.05, **p<0.005, ***p<0.0005.

LINC00460 over-expression was significantly associated with advanced and locally advanced tumor clinical stages in three distinct cancers: LUSC, LUAD and KIRC (ANOVA; p<0.05; **Supplementary Figures S3A–C**). In the KIRC model, LINC00460 over-expression is significantly associated with high histological grade (Mann Whitney U-test; p=0.0001; **Supplementary Figure S3D**). In addition, LINC00460 overexpression is correlated with further aggressive tumor phenotypes, such as HPV negative HNSC (TCGA: Mann-Whitney U, p< 0.05. GSE3292: one-way ANOVA, p= 0.02) and basal-like BRCA (TCGA: Kruskal-Wallis test, p=3.14E-13. Mexican cohort: Kruskal-Wallis test, p=0.012), using two independent cohorts for each cancer type (**Supplementary Figure S4** and **Figures 2A and B**). This last observation was further validated in a panel of BRCA cell lines, where we detected that LINC00460 is mainly expressed in basal TNBC cells (**Figure 2C**). CCLE screening confirmed TNBC LINC00460 enrichment (**Supplementary Figure S6**). As LINC00460 expression is significantly related to Basal-like BRCA, we reasoned that LINC00460 over-expression might also be associated with Immunohistochemistry (IHC)-detected Estrogen Receptor (ER) and Progesterone Receptor (PR) status in BRCA patients. As expected, LINC00460 expression is enriched in IHC-detected negative Estrogen receptor (ER) (Mann-Whitney U; p=0.000079) and Progesterone Receptor (PR) (Mann-Whitney U; p= 0.024) TCGA BRCA patients (**Figure 2D**). These observations were validated in an independent mexican cohort (Chi squared test ER p-value= 0.028; Chi squared test PR p-value=0.001) (**Figure 2E**).

### LINC00460 Is Related With Poor Prognosis in Eight Different Tumors, but Increased Survival Rate in BRCA

**Figure 3:**
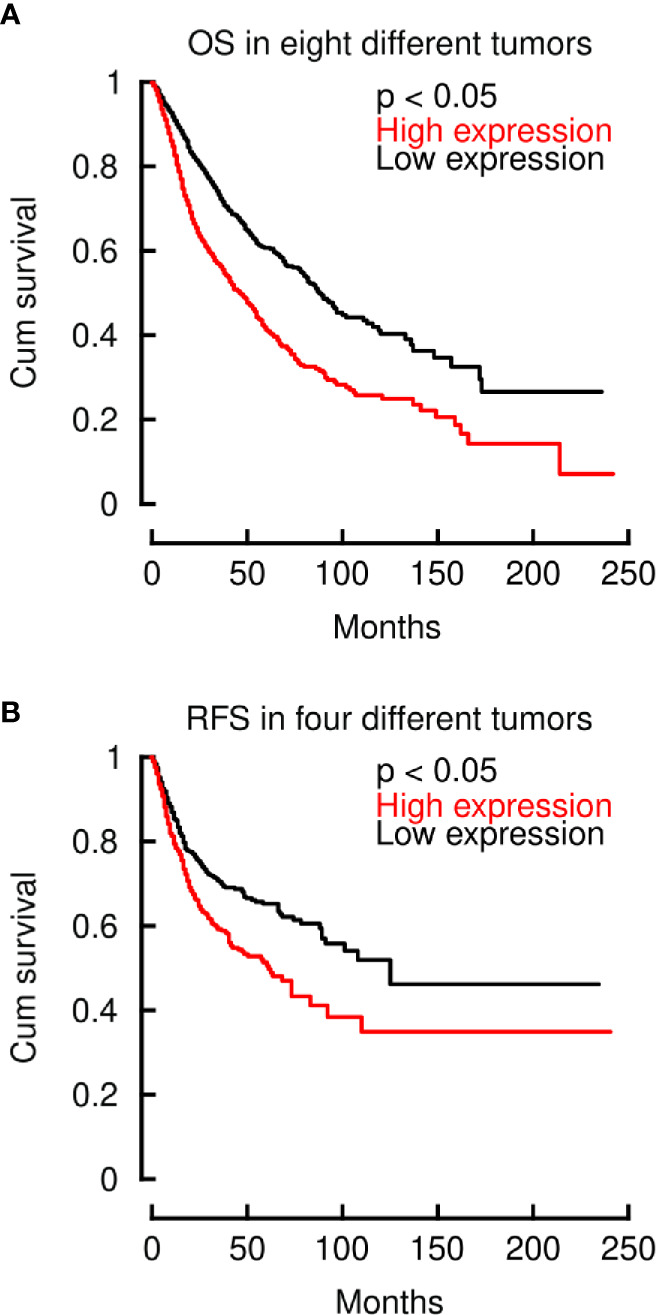
LINC00460 deregulation is related to high risk of decease and relapse across multiple cancers. **(A)** LINC00460 overexpression is significantly associated with elevated HR of decease in eight different tumors (CESC, GBM, HNSC, KIRC, LGG, LUAD, PAAD and SARC). **(B)** High LINC00460 expression is associated with increased risk of relapse in four different tumors (KIRC, LUAD, READ and SARC).

After demonstrating that LINC00460 is significantly related to aggressiveness markers in different tumors, we then aimed to know if its deregulation is also related to overall survival (OS) and relapse free survival (RFS) in these cancers, using the TCGA cohorts. As shown in **Figure 3**, LINC00460 overexpression is significantly related to high risk of death in eight different tumors, analyzed in a single model (CESC, GBM, HNSC, KIRC, LGG, LUAD, PAAD and SARC) (log2HR>1; p<0.05) (**Figure 3A**), and with high risk of relapse in four cancers (KIRC, LUAD, READ and SARC) (log2HR>1; p<0.05) (**Figure 3B**). Taken together with previous findings reported in the field (30, 34, 47, 48, 50–52, 54, 55, 57), this data suggests a relevant role for LINC00460 in clinical cancer biology.

**Figure 4:**
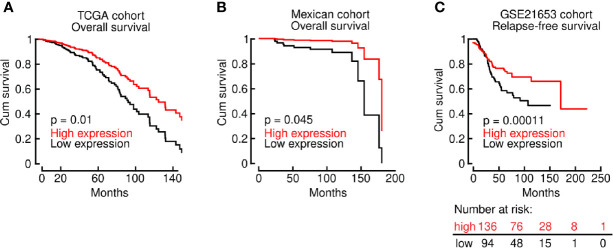
LINC00460 up-regulation is associated with improved OS and RFS in multiple BRCA cohorts. **(A)** LINC00460 over-expression is significantly associated with improved OS in the BRCA TCGA cohort; **(B)** in an independent mexican patient cohort and **(C)** the GSE21653 GEO dataset.

An unexpected observation in this regard is the effect of LINC00460 over-expression in BRCA, which is associated with improved OS (**Figure 4A**), compared with the LINC00460 low expression group in two independent cohorts, namely TCGA (through Tanric tool, n=743; logrank p=0.01) and mexican (n=93; HR=1.655; 95% CI [1.038-26.41]; logrank p=0.045) (**Figure 4B**). The clinical and pathological characteristics of the TCGA and mexican BRCA patients are described in **Table 1**.

**Table 1:** Clinicopathological characteristics of the mexican BRCA cohort (n = 107).

Variable	Stratification Frequency n				
Age	<50	>50	NA		
	31	69	7		
ER	Negative	Positive	NA		
	35	68	4		
PR	Negative	Positive	NA		
	47	56	4		
HER2	Negative	Positive	NA		
	79	23	5		
Clinical Stage	I	II	III	IV	
	27	64	9	7	
Grade	I	II	III	NA	
	14	64	20	9	
Defunction	Positive	Negative	NA		
	11	94	2		
Metastasis	Positive	Negative	NA		
	9	26	72		
Survival (months)	< 60	>60	NA		
	16	91	0		
Tumor size	<20mm	>21 a 49 mm	>50 mm	NA	
	33	49	9	16	
Molecular subtype (IHC)	Luminal A	Luminal B	Her2	Basal	NA
	44	25	11	17	10
Lymphocyte infiltration	≤50	≥50	NA		
	61	1	22		

In addition, we observed that LINC00460 expression level statistically interacts with PR, HER2 status, patient age, and tumor grade in the survival Cox regression analysis of the Mexican cohort (**Supplementary Table S2**). These interactions suggest that LINC00460 is involved in important cancer-related processes like tumor differentiation, hormonal status and HER2 expression in mexican BRCA patients, although the exact related mechanisms remain unclear.

We have also assessed RFS in the GSE21653 dataset (n=240; HR = 0.59; 95% CI [0.38 − 0.94]; logrank p = 0.024) and DMFS (n=120; HR=0.78; 95% CI [0.6-1.02]; logrank p= 0.062) in BRCA patients in the GEO-derived cohorts (**Figure 4C** and **Supplementary Figure S5**). We found a corresponding significant association with improved RFS for LINC00460-high patients. Although non-significant, we observed a solid tendency to a higher DMFS in the BRCA LINC00460-overexpressed group (**Supplementary Figure S5**).

These data strongly suggest that LINC00460 might play a dual prognostic role across different tumors, as high LINC00460 expression predicts an increased OS, RFS and DMFS in the BRCA model, but it is also a marker for poor prognosis in at least eight distinct solid tumors (see **Figure 3C**). These intriguing findings aimed us to further investigate the role of LINC00460 in BRCA.

### LINC00460 Is Significantly Enriched in Basal-Like 2 TNBC and Its Overexpression Predicts a Favorable Clinical Course

**Figure 5:**
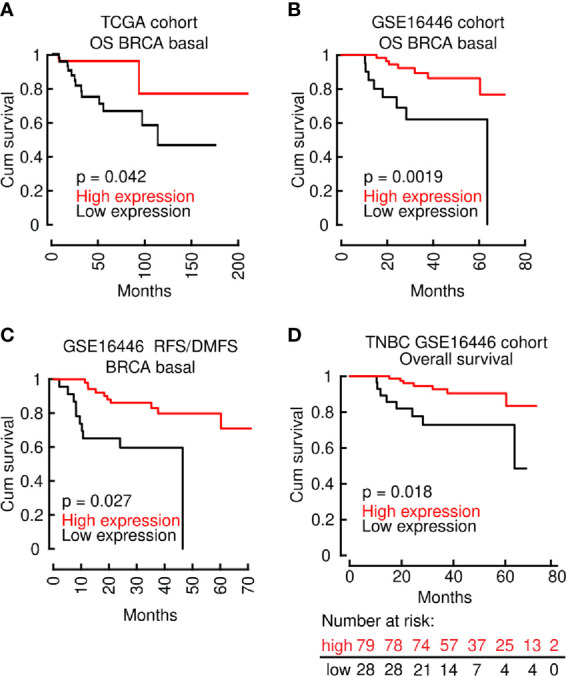
The effect of improved OS, RFS and DMFS in the LINC00460-high group is maintained in Basal type BRCA. **(A)** OS in the TCGA BRCA Basal cohort; **(B)** OS in the GSE16446 BRCA Basal cohort; **(C)** RFS/DMFS in the GSE16446 BRCA Basal cohort; **(D)** OS in the GEO TNBC cohort.

We then directed our efforts to elucidate the role of LINC00460 in OS prediction of the aggressive Basal-like BRCA model, a subtype which comprises the majority of TNBC cases (77). Interestingly, the improved OS effect shown in all BRCA patients (**Figures 4A, B**), is reproduced when we analyzed two independent cohorts of Basal-like BRCA tumors only, namely the TCGA cohort (TCGA *via* Tanric tool, n=139; logrank p=0.042) and GEO derived cohort GSE16446 (using basal-like samples only; n= 76; HR=0.23[0.08−0.63]; logrank p = 0.0019) (**Figures 5A, B**). RFS/DMFS (GSE16446; n= 76; HR = 0.27; [0.11 − 0.68]; logrank p = 0.0027) were also significantly improved in LINC00460 over-expressed BRCA basal-like samples (**Figure 5C**). Analysis with LINC00460 predicted an increased OS in the TNBC GEO derived cohort GSE16446 (using all TNBC samples; n= 107; HR = 0.26; 95% CI [0.09 − 0.72]; logrank p = 0.0053) (**Figure 5D**).

**Figure 6:**
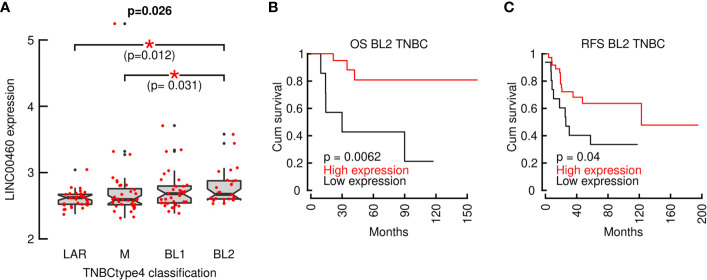
LINC00460 is enriched in Basal-like 2 TNBC (TNBCtype4) and its expression can predict improved OS and RFS. **(A)** LINC00460 is significantly enriched in BL2 TNBC; **(B)** LINC00460 over-expression in associated with increased OS and **(C)** RFS in BL2 TNBC. *p<0.05.

To further characterize LINC00460 association with aggressive BRCA, we analyzed its expression in Lehman refined triple negative breast cancer classification (TNBCtype4) (**Figure 6A**). We observed a significant LINC00460 enrichment in the Basal-like 2 (BL2) subtype (n= 135, Jonckheere-Terpstra test for ordered variables; p=0.026) in a Chinese BRCA cohort (GSE76250). LINC00460 high expression level is also able to predict a favorable clinical response in BL2 triple negative BRCA GEO cohorts, both in OS (n=28; logrank, p=0.0062; **Figure 6B**) and RFS (n= 52; logrank, p=0.04; **Figure 6C**).

Altogether, these observations suggest that LINC00460 expression is generally related to intrinsically aggressive tumor phenotypes, as shown for HNSC, LUSC, LUAD, KIRC and basal-like BRCA (**Figure 2** and **Supplementary Figures S3, 4**). These findings are further corroborated when we showed that LINC00460 is enriched in the TNBCtype4 BL2 subtype in an independent cohort (**Figure 6A**). Interestingly, in aggressive BRCA subtypes, high LINC00460 expression is able to predict a favorable clinical course, further strengthening the dual role for this lncRNA in OS and RFS prediction in cancer.

### LINC00460 Potentially Regulate a Plethora of Cancer-Related Genes in BRCA Involved in Proliferation, Cell Cycle and Migration

**Figure 7:**
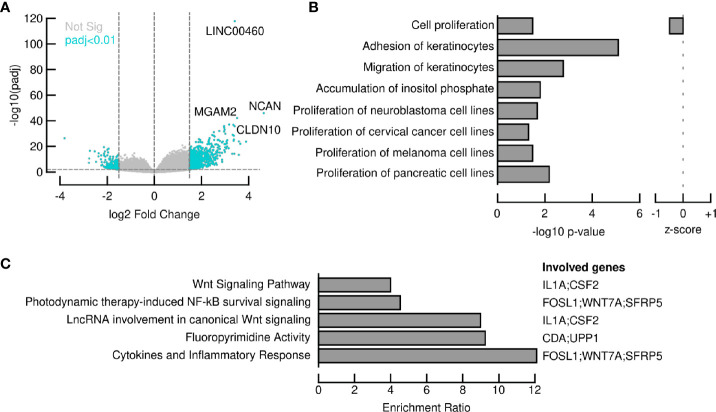
LINC00460 potentially regulates WNT signaling pathway and the cytokine inflammatory response in BRCA. **(A)** 874 transcripts were significantly deregulated after comparing LINC00460-high samples vs LINC00460-low samples; **(B)** Several cancer-related pathways, such as cell proliferation, cellular migration, adhesion, cell cycle progression and carbohydrate metabolism are potentially altered in LINC00460-high BRCA samples; **(C)** ORA revealed LINC0460 involvement in WNT signaling and cytokine inflammatory response among other pathways in BRCA samples.

In order to elucidate the intriguing role of LINC00460 in BRCA, we aimed to identify potential LINC00460 expression targets. Differential expression analysis was performed in TCGA BRCA samples between the LINC00460 high expression group and the LINC00460 low-group (see methods for details). This approach revealed 874 significantly deregulated transcripts (FC<1.5 and <-1.5, p.adj value<0.01; **Figure 7A**). Of those, 73% (638 RNAs) were up-regulated, and the remaining were found down-regulated (27%; 236 transcripts) between groups. Among the up-regulated RNAs, we identified several cancer-related transcripts, such as *HOXD13, CXCL1, CXCL5, FOXG1, SERPINB4, CLDN6* and *CLDN10* (see **Table 2**). Down-regulated transcripts list includes *EMX1, CYP2G1P, AMELX* and *SOX5-AS1* (**Table 3**). Furthermore, Ingenuity Pathway Analysis revealed that the proliferation process was negatively enriched in the high LINC00460 group (z score= -0.513). Cellular migration, adhesion, cell cycle progression and proliferation were also enriched in these BRCA samples, although no z-score enrichment was detected in these pathways (see **Figure 7B**).

**Table 2:** LINC00460 high vs low up-regulated genes.

	Gene symbol	Gene name	log2FC	padj-value
ENSG00000130287	NCAN	Neurocan	4.7	5.41E-50
ENSG00000103355	PRSS33	Serine Protease 33	3.7	1.22E-24
ENSG00000140522	RLBP1	Retinaldehyde binding protein 1	3.7	4.03E-27
ENSG00000275216	AL161431.1		3.7	8.28E-15
ENSG00000112238	PRDM13	PR Domain-containing protein 13	3.5	9.31E-17
ENSG00000177359	AC024940.2		3.4	2.06E-38
ENSG00000233532	LINC00460		3.4	1.00E-126
ENSG00000134760	DSG1	Desmoglein 1	3.4	4.23E-31
ENSG00000224887	AL513318.1		3.4	4.13E-16
ENSG00000166535	A2ML1	Alpha-2-Macroglobulin-Like 1	3.3	4.23E-31
ENSG00000206073	SERPINB4	Serpin Family B member 4	3.3	7.15E-15
ENSG00000181617	FDCSP	Follicular dendritic cell secreted protein	3.2	3.44E-21
ENSG00000229921	KIF25-AS1	KIF25 antisense RNA 1	3.2	2.85E-24
ENSG00000163735	CXCL5	C-X-C motif chemokine ligand 5	3.2	8.62E-34
ENSG00000047936	ROS1	ROS proto-oncogene 1	3.2	8.15E-28
ENSG00000134873	CLDN10	Claudin 10	3.2	2.36E-39
ENSG00000128714	HOXD13	Homebox D13	3.0	7.62E-27
ENSG00000163739	CXCL1	C-X-C motif chemokine ligand 1	3.0	1.66E-36
ENSG00000184697	CLDN6	Claudin 6	2.9	1.62E-25
ENSG00000176165	FOXG1	Forkhead box G1	2.7	2.22E-13

**Table 3:** LINC00460 high vs low down-regulated genes.

	Gene symbol	Gene name	log2FC	padj-value
ENSG00000234398	AC134915.1		-1,5	0,00659454174
ENSG00000236532	LINC01695		-1,5	6,95E-09
ENSG00000137948	BRDT	Bromodomain Testis associated	-1,5	1,66E-07
ENSG00000238391	RNA5SP233	5S ribosomal pseudogene 233	-1,5	0,003359924242
ENSG00000275251			-1,5	0,004665490892
ENSG00000249691	AC026117.1		-1,5	0,003145246539
ENSG00000130612	CYP2G1P	Cytochrome P450 family 2 subfamily G member 1, pseudogene	-1,5	7,07E-07
ENSG00000125363	AMELX	Amelogenin X-Linkes	-1,5	0,005986652631
ENSG00000135638	EMX1	Empty spiracles homebox 1	-1,5	2,26E-07
ENSG00000256120	SOX5-AS1	GRIK1 antisense-RNA1	-1,5	0,009555483978
ENSG00000255155	AP004371.1		-1,6	1,77E-06
ENSG00000225795	AC006463.1		-1,6	0,004581123508
ENSG00000256612	CYP2B7P	Cytochrome P450 family 2 subfamily B member 7	-1,6	2,64E-06
ENSG00000265595	MIR4756		-1,6	0,001510008292
ENSG00000186732	MPPED1	Metallophosphoesterase domain containing 1	-1,6	1,43E-08
ENSG00000248350	AC010265.1		-1,6	0,004980797457
ENSG00000139352	ASCL1	Achaete-scute family bHLH transcription factor 1	-1,6	3,46E-05
ENSG00000233420	AC002127.2		-1,6	0,001874852522
ENSG00000250223	LINC01216		-1,6	0,007490194855

This data pinpoints the role of LINC00460 as a potential regulator of transcripts and cellular cancer-related processes like proliferation, migration and cell proliferation in BRCA.

### LINC00460 Expression Is Significantly Correlated With the WNT Pathway and Cytokine Inflammatory Response Genes

LINC00460 expression level is correlated with at least 100 coding transcripts in BRCA (PCC>0.45, p<0.05; shown in **Table 4**). Some of these transcripts are classic cancer-related, such as *WNT7A, SFRP5, FOSL1, IFNK, CSF2, DUSP7* and *IL1A*. Overrepresentation Enrichment Analysis (ORA) showed that WNT signaling pathway, cytokine inflammatory response, fluoropyrimidine activity pathway and photodynamic therapy-induced NF-kB survival signaling are enriched in LINC00460 over-expressed BRCA samples (**Figure 7C**). In addition, we observed that 73% (61/84) of the BRCA Mexican cohort samples displayed high levels of stromal TILs (see **Table 1**). Analyzing the LINC00460 co-expressed genes list, we observed that similar genes had been previously identified as enriched in Lehman’s TNBC subtypes (77) (**Supplementary Table S3**). Altogether, these enrichment data suggests that LINC00460 is potentially related with inflammatory pathways and might partially explain its effect in good prognosis prediction in BRCA.

### LINC00460 Is an In-Silico Predicted miR-103-a-1 Sponge

LINC00460 has been described as a miRNA sponge lncRNA in different tumors (38, 44, 60, 75). With this evidence in mind, we aimed to further characterized its biological role in BRCA. We used an *in-silico* approach to predict LINC00460 interaction with novel miRNAs, using the miRcode tool (**Table 4**).

In the former section, we found that a group of mRNAs are significantly correlated with LINC00460 levels (see **Table 4**). We then reasoned that, if LINC00460 is acting as a miRNA sponge as previously reported, then some of these correlated mRNAs may be miRNAs targets as well. We used two *in-silico* tools to predict mRNA-miRNA binding, namely miRcode and mirPATHDB. As shown in **Table 5**, the 10 LINC00460 most correlated mRNAs have potential miRNA interactants. Furthermore, we observed that some of these mRNAs, such as WNT7A and KRTAP11-1, can potentially bind to the same miRNA: miR-103-a-1 (**Tables 5** and **6**). These findings suggest a role for LINC00460 as a miR-103-a-1 interactant and as a potential regulator of WNT7A and KRTAP11-1 expression.

**Table 4:** LINC00460 correlated genes.

	Gene symbol	Gene name	PCC*
ENSG00000179046.8	TRIML2	Tipartite motif family like 2	0.61
ENSG00000175592.8	FOSL1	FOS like 1, AP-1 transcription factor subunit	0.6
ENSG00000115008.5	IL1A	Interleukin 1 alpha	0.57
ENSG00000163915.7	IGF2BP2-AS1		0.56
ENSG00000164400.5	CSF2	Colony stimulating factor 2	0.55
ENSG00000240476.1	LINC00973		0.55
ENSG00000147896.3	IFNK	Interferon kappa	0.54
ENSG00000154764.5	WNT7A	Wnt family member 7A	0.54
ENSG00000261780.2	CTD-2354A18.1		0.53
ENSG00000254403.1	OR10Y1P	Olfactory receptor family 10 subfamily Y member 1 pseudogene	0.53
ENSG00000158825.5	CDA	Cytidine deaminase	0.53
ENSG00000104327.7	CALB1	Calbindin 1	0.51
ENSG00000203783.4	PRR9	Proline rich 9	0.51
ENSG00000176797.3	DEFB103A	Defensin beta 103A	0.48
ENSG00000182591.5	KRTAP11-1	Keratin associated protein 11-1	0.48

*PCC, Pearson correlation coefficient.

**Table 5:** Matching sites and predicted miRNAs interacting with LINC00460.

microRNA family	Seed position	Seed type
miR-503	chr13:107029280	7-mer-m8
miR-143/1721/4770	chr13:107028970	7-mer-m8
miR-150/5127	chr13:107029727	7-mer-m8
miR-1ab/206/613	chr13:107030346	7-mer-A1
miR-200bc/429/548a	chr13:107029706	7-mer-A1
miR-221/222/222ab/1928	chr13:107029417	7-mer-A1
miR-23abc/23b-3p	chr13:107029820	7-mer-A1
miR-24/24ab/24-3p	chr13:107029217	7-mer-A1
miR-24/24ab/24-3p	chr13:107029855	7-mer-A1
miR-24/24ab/24-3p	chr13:107029953	8-mer
miR-103a/107/107ab	chr13:107030479	8-mer
miR-338/338-3p	chr13:107029282	7-mer-A1
miR-338/338-3p	chr13:107029426	7-mer-A1
miR-425/425-5p/489	chr13:107029977	7-mer-A1
miR-129-5p/129ab-5p	chr13:107030286	7-mer-A1

**Table 6:** Predicted miRNAs that are potentially interacting with LINC00460 and some coexpressed mRNAs.

miRNA	mRNA	PCC (mRNA vs LINC00460)
miR-133a-3p	TRIML2	0.61
miR-130a	FOSL1	0.6
miR-544b	CSF2	0.55
miR-216b	IFNK	0.54
miR-103a-1	WNT7A	0.54
miR-34a	CDA	0.53
miR-140-5p	CALB1	0.51
miR-124-1	DEFB103A	0.48
miR-103a-1	KRTAP11-1	0.48
miR-455-5p	FERMT1	0.47

### The LINC00460: WNT7A Ratio Is a Composite Marker for Increased OS and DMFS in Basal-Like BRCA and Can Predict Anthracycline Response in ER- BRCA Patients

**Figure 8:**
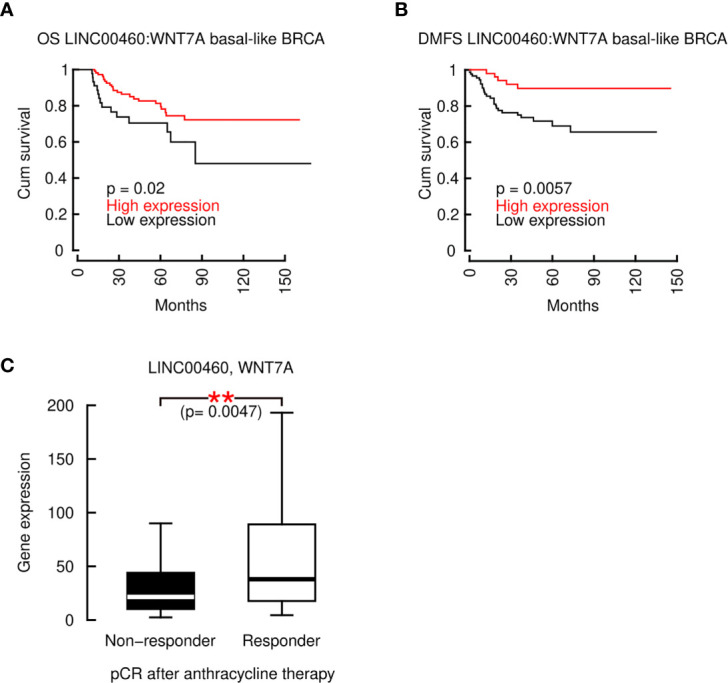
The composite marker LINC00460:WNT7A is predicts an increased OS and RFS in basal-like BRCA and its median is associated with a complete pathological response to chemotherapy in ER- BRCA patients. **(A)** LINC00460:WNT7A ratio predicts a significantly improved OS and **(B)** RFS in basal-like BRCA; **(C)** LINC00460 and WNT7A combined expression is significantly associated with pCR after anthracycline-based therapy in ER- patients. **p<0.005.

To further demonstrate the role of LINC00460 in potential regulation of WNT7A and its combined role in BRCA prognosis, we computed the LINC00460:WNT7A ratio to construct an OS and DMFS models for Basal-like BRCA. As shown in **Figure 8**, the LINC00460:WNT7A ratio is able to predict an increased OS (n=153, logrank p=0.028) and DMFS (n=145, logrank p=0.0057) in Basal-like BRCA, using GEO cohorts. In contrast, analysis with LINC00460:KRTAP11-1 ratio did not retrieve any significant survival effect (data not shown). Survival analysis using the mature sequence of miR-103-a-1 (hsa-miR-103a), showed a marginal, non-significant association between overexpression of the of miR-103-a-1 and decreased survival in TCGA TNBC cohort (n=97, logrank p=0.059; **Supplementary Figure S7**).

Taken together, these data show that the expression ratio of two genes, LINC00460:WNT7A is a composite marker that accurately predicts Basal-like BRCA OS and DMFS. Furthermore, we identified that the combination of LINC00460 and WNT7A over-expression is significantly associated with pathological complete response (pCR) after anthracycline therapy in ER- BRCA patients (n= 665, Mann-Whitney U test p= 0.0047) (**Figure 8C**). These evidences clearly indicate that both transcripts exert a central and beneficial role to basal-like, ER- BRCA patients.

## Discussion

Long non-coding RNAs exert numerous roles in human cancers, as their biological activities involve regulation of cell proliferation, cell death, differentiation, migration, invasion and metastasis. Deregulation in lncRNAs expression has also been associated with clinical outcome. LncRNAs can affect expression of thousands of genes, so they are regarded as key master regulators (78).

In this work, our aim was to investigate if LINC00460 expression was deregulated in different tumors, and if it was associated with clinical and pathological characteristics in these tumors. We then focused on its clinical role in aggressive (basal-like) breast cancer and the identification of potential LINC00460 targets in this model. We aimed to know if LINC00460 can target miRNAS *in-silico*, which can in turn bind to relevant mRNAs. Finally, we sought to investigate if some of the potential candidate genes would have a combinatorial role for OS and therapy response in basal-like BRCA.

We found that LINC00460 was deregulated in seven different tumor types in the TCGA database, including two not previously reported tumors, namely LGG and GBM. We have also confirmed LINC00460 deregulation in BRCA using two independent cohorts. Interestingly, LINC00460 expression is associated with clinically aggressive tumors, such advanced stage LUAD, LUSC and KIRC, high histology grade in KIRC, HPV-negative HNSC and basal-like BRCA, suggesting an important role of LINC00460 in the progression or intrinsically aggressiveness of these tumors. Indeed, it has been previously shown that LINC00460 expression can promote cancer progression (34, 35, 38, 50), metastasis (36) and influences therapy response (79).

Several reports show that LINC00460 is a marker for poor OS prognosis across different tumors, such as CESC (48), HNSC (80), KIRC (81), LUAD (49) and PAAD (82). In addition, we describe here that LINC00460 high expression is significantly associated with poor survival in three different tumors (GBM, LGG and SARC) but related with a favorable survival rate in BRCA, *i.e.*, its association to clinical outcome varies between tumors. This data suggests that the expression levels and its impact on OS, RFS or DMFS may be tissue-specific. Indeed, it has previously been shown that the same lncRNA may exert dual prognostic roles in distinct tumors. For example, high MALAT-1 expression has been reported as a marker for poor prognosis in various tumors, including COAD, NSCLC, STAD, PAAD, ESCA, among others (83), but also as a good prognosis factor for BRCA, acting as a metastasis suppressor (84). Another interesting example of this dual phenomenon is the expression of XIST. It has been shown that high expression of this lncRNA is related to poor clinical outcome in different cancers (85), but in another study, authors demonstrate that high XIST expression is related to an increased brain metastasis–free survival in BRCA patients (6). We suggest that LINC00460 can perform as a dual tissue-specific prognostic marker, similar to MALAT-1 and XIST; although we cannot discard alternative mechanisms, such as differences in the splicing variants measured between studies. These variant transcripts expression patterns should be taken into account to evaluate lncRNA-based predictive biomarkers (86). This latter possibility must be addressed in future studies.

In the BRCA model, LINC00460 expression is associated with the phenotypic makeup of the tumor, where overexpression of LINC00460 is associated with a negative result to hormone receptors (ER and PR) when subject to IHC. It was also observed that in the most aggressive phenotype, basal-like BRCA, it actually favored the clinical outcome (**Figure 6**), even when being subject to analysis in the different TNBC subtypes. Its similar behavior through different BRCA subtypes and BRCA different cohorts, led us to propose LINC00460 as a potential biomarker for improved OS, RFS and conceivably DMFS prediction in basal-like BRCA (**Figures 4–6**). This behavior can potentially be explained through the candidate co-expressed genes found in the differential expression analyses and ORA (**Figure 7**). We propose five main mechanisms that potentially explain the role of LINC00460 in the increased prognosis of BRCA patients: (1) regulation/co-expression of good prognosis-related genes, (2) co-expression/modulation of genes that promote an immunogenic niche, (3) decrease of tumor cell proliferation, (4) regulation of WNT7A through sponging of miR-103-a-1 and (5) promoting chemotherapy (anthracycline) complete pathologic response. As the majority of these interactions are *in-silico* predicted, or associated with clinical features in patient-based cohorts, experimental validation is needed to support these hypotheses.

In this regard, we observed that the LINC00460 expression is significantly enriched in the BL2 TNBC subtype. This finding is of particular interest, since the BL2 subtype displays a variety of gene ontologies enriched in components and pathways involved in cell proliferation, growth, survival and cell differentiation pathways, such as the WNT pathway (77). In accordance, we identified important members of the WNT pathway, such as WNT7A, as being potentially regulated by LINC00460 (see **Figure 7C** and the discussion below). These data strengthen the role of LINC00460 in BL2 TNBC, and further clarify its mechanism of action in these tumors.

We detected that some of the LINC00460 co-expressed genes, such as *SFRP5, HOXD13*, are also related to increased survival rate in different tumors. High expression of *SFRP5* is significantly associated with a better prognosis in PAAD (87) and BRCA (88). HOXD13 protein levels are related to an increased OS in BRCA (89). Thus, this LINC00460 candidate target genes potentially contribute to the increased survival effect observed in BRCA patients.

On the other hand, we found that several LINC00460 co-expressed genes (*TRIML2*, *SFRP5, FOSL1, IFNK, CSF2, DUSP7, DEFB103A* and *IL1AA)* are immunogenic-related. Immune response pathways are clinically relevant, as it has been previously described that a highly immunogenic niche in a tumor may improve the outcome of the disease (90). These genes and pathways are frequently enriched in TNBC (77, 91). Furthermore, TNBC also display enrichment of tumor-infiltrating lymphocytes (TILs) (92). We suggest that LINC00460 is related with good prognosis in TNBC/basal-like due to its potential relation with these genes and with the presence of tumor-infiltrating lymphocytes (TILs), as shown in our samples (see **Table 1**). It has been previously shown that the presence of TILs improves prognosis as it modulates cancer progression and enhances chemotherapy response in TNBC, conferring a protective immunity in these patients (93).

Therefore, we suggest that the correlation between these immunogenic genes and LINC00460 can partially explain the clinical behavior in the breast cancer cohorts, as the overexpression of LINC00460 is associated with upregulation of immunogenic factors that in turn, permit the migration of components of the immune system. These mechanisms can promote an immunogenic tumor environment and thus favor tumor cell death. Future studies are needed to experimentally validate these data, as it is relevant for clinical outcome in aggressive TNBC.

In addition to promoting the immunogenic niche, LINC00460 could decrease proliferation of the tumor cells, as we observed that the proliferation pathway is negatively enriched in the high LINC00460 group in BRCA samples. This could also explain the increased survival rates of the BRCA patients. Indeed, multiple lines of evidence suggest that LINC00460 can modulate cell proliferation, cell death, migration and invasion and EMT, through its sponging activity and targeting various key transcripts in several cancer types (31, 37, 43, 94–96).

We predicted that LINC00460 potentially binds to miR-103-a-1 *in-silico*, which, in turn, can target WNT7A. In this regard, although there have been reports that suggest that WNT7A is an oncoprotein (97), it has also been shown that loss of WNT7A expression is significantly associated with poor RFS in BRCA (98) and it is also involved in tumor cell differentiation (99). Thus, the exact role of WNT7A in BRCA is currently unclear. Our results might suggest that WNT7A potentially play an important clinical role in the BRCA, as LINC00460 could sponge miR-103-a-1 and henceforth, liberate WNT7A. Further research must validate these predictions experimentally.

We observed that the LINC00460:WNT7A ratio is a composite marker that can predict a favorable OS and DMFS in TNBC. These results highlight the clinical and biological role of LINC00460 and WNT7A transcripts in TNBC and constitute valuable data, as simple ratios of gene expression levels can be used to accurately diagnose (100) and predict cancer outcomes (101, 102), while circumventing many of the limitations that preclude the use of microarray techniques in extensive clinical applications (103, 104). In accordance with our observations regarding immunogenic factor potential upregulation and previous findings in the field (92, 93, 105), we have also identified a significant association between the expression enrichment of LINC00460 and WNT7A with anthracycline responsive ER- patients. This finding further strengthens the beneficial role of both transcripts in patient’s prediction and prognosis as, it has been demonstrated that ER-negative breast cancers with high levels of TILs have heightened sensitivity to anthracycline-based chemotherapy (106), and that TILs are an independent predictor of good response to anthracycline/taxane neoadjuvant chemotherapy (105). All these observations, however, are limited by the size of GEO patient cohorts and will require validation in a larger independent cohort.

Regarding the role of miR-103-a-1, we identified a marginal association with poor OS in BRCA. This is in accordance with previous findings, as it has been shown that miR-103-a-1 acts as an oncogene to promote TNBC cells migration and invasion (107). In another report, authors show that serum miR-103 over-expression was significantly correlated with worse clinical factors, as well as poorer recurrence-free survival or overall survival in colorectal cancer (108). MiR-103/107 expression displays stemness-promoting functions, and a signature of miR-103/107 high and Axin2 low expression profile correlates with poor prognosis in colorectal cancer patients (109). In gastric cancer patients, high expression of miR-103 was significantly associated with poor overall survival and disease-free survival and is a key factor that contributes to tumor progression (110). Altogether, these data suggest that of miR-103-a-1 is a marker for poor prognosis in several tumors, including BRCA. To the best of our knowledge, this is the first report that suggests a potential connection between LINC00460, WNT7A and miR-103-a-1. Future research must elucidate the exact mechanisms involved in this potential 3-gene network, and its impact in basal-like BRCA biology.

In conclusion, LINC00460 expression is a dual potential marker for aggressive phenotypes and poor clinical outcome in distinct tumors, including HNSC, KIRC LUSC and LUAD, that is also associated with increased prognosis in basal-like BRCA. LINC00460 is enriched in BL2 TNBC, and potentially regulates the WNT differentiation pathway. LINC00460 can also modulate a plethora of immunogenic related genes in BRCA, such as *SFRP5, FOSL1, IFNK, CSF2, DUSP7* and *IL1A* and interacts with miR-103-a-1, *in-silico*, which, in turn, can no longer target WNT7A. LINC00460:WNT7A ratio is a composite marker that can predict a favorable OS and DMFS in TNBC, and combination of LINC00460 and WNT7A over-expression is associated with complete pathological response (pCR) after anthracycline therapy in ER- BRCA patients. This data confirms that LINC00460 is a master regulator in BRCA molecular circuits and influences clinical outcome.

## Data Availability Statement

The original contributions presented in the study are included in the article/**Supplementary Material**. Further inquiries can be directed to the corresponding authors.

## Ethics Statement

The studies involving human participants were reviewed and approved by Research and Ethics Committee of National Institute of Genomic Medicine and the Institute of Breast Diseases, FUCAM (Registration number: CE2009/11). The patients/participants provided their written informed consent to participate in this study.

## Author Contributions

AH-M, MR-R, LH-P and MC-V conceived and designed the study. MM-R and LH-P handled the samples, constructed the clinical database and tested the expression levels in biological samples. SJ-M, RA-L, LA-R, and LH-P handled the samples and clinical database. AT-T, CD-R, FV-C, IR-S, and AM-A obtained the samples and performed the clinical evaluation and following of the patients. LH-P did the pathological evaluation of the samples. AC-T, MC-V, MP-L, RA-L, and EH-C performed all the *in-silico* validation cohort-related analysis and the *in-silico* prediction of the sponge regulation. MR-R, MC-V, and AC-T performed statistical analysis, survival analysis, differential expression analysis, and risk analysis. All authors interpreted and discussed the data. All authors contributed to the article and approved the submitted version.

## Funding

This work was funded by the Mexican National Council of Science and Technology 480751/282036 grant (Scholarship), and Mexican National Council of Science and Technology Basic Science grant (CONACYT grant number 258936).

## Conflict of Interest

The authors declare that the research was conducted in the absence of any commercial or financial relationships that could be construed as a potential conflict of interest.
