# Supplementary material for: LINC00460 Is a Dual Biomarker That Acts as a Predictor for Increased Prognosis in Basal-Like Breast Cancer and Potentially Regulates Immunogenic and Differentiation-Related Genes
Source: Front Oncol. 2021 Apr 12;11:628027. doi: 10.3389/fonc.2021.628027 (PMC8074675; doi:10.3389/fonc.2021.628027)
Supplement: Supplementary file 1 [file DataSheet_1.docx]

Supplementary Material

Supplementary Table S1. TCGA cancer types and sample size for tumors and correspondent normal tissues

| Cancer type | Sample size | |
| --- | --- | --- |
|  | ***Tumor*** | ***Normal*** |
| ACC | 77 | 128 |
| BLCA | 404 | 28 |
| BRCA | 1085 | 291 |
| CESC | 306 | 13 |
| CHOL | 36 | 9 |
| COAD | 275 | 349 |
| DLBC | 47 | 337 |
| ESCA | 181 | 286 |
| GBM | 163 | 207 |
| HNSC | 519 | 44 |
| KICH | 66 | 53 |
| KIRC | 523 | 100 |
| KIRP | 286 | 60 |
| LAML | 173 | 70 |
| LGG | 518 | 207 |
| LIHC | 369 | 160 |
| LUAD | 483 | 347 |
| LUSC | 486 | 338 |
| MESO | 87 | NA |
| OV | 426 | 88 |
| PAAD | 179 | 171 |
| PCPG | 182 | 3 |
| PRAD | 492 | 152 |
| READ | 92 | 318 |
| SARC | 262 | 2 |
| SKCM | 461 | 558 |
| STAD | 408 | 211 |
| TGCT | 137 | 165 |
| THCA | 512 | 337 |
| THYM | 118 | 339 |
| UCEC | 174 | 91 |
| UCS | 57 | 78 |
| UVM | 79 | NA |

**Supplementary Table S2. Variables in the Equation for Cox Regression Analysis (Mexican cohort)**

|  | **B** | **SE** | **Wald** | **Sig.** | **Exp (B)** | **95%CI** |  |
| --- | --- | --- | --- | --- | --- | --- | --- |
|  |  |  |  |  |  |  |  |
| **LINC00460 expression (median)** | 1.655 | .826 | 4.020 | .045 | 5.235 | 1.038 | 26.408 |
| **Clinical Stage** | .646 | .342 | 3.571 | .059 | 1.908 | .976 | 3.728 |
| **Tumor Grade** | -.639 | .950 | .453 | .501 | .528 | .082 | 3.397 |
| **PR status** | 1.430 | .731 | 3.828 | .050 | 4.178 | .997 | 17.501 |
| **HER2 status** | -1.416 | .689 | 4.224 | .040 | .243 | .063 | .936 |
| **Patient age** | .320 | .855 | .141 | .708 | 1.378 | .258 | 7.355 |

**Supplementary Table S3. Expression levels of genes that correlate with LINC00460 expression in TNBC/basal-like and its relationship with similar genes in TNBC subtypes.**

| LINC00460 correlation | GEPIA2 expression  Basal-like / Triple negative (135) | Differentially expressed genes in TNBC Lehmann et al., 2011 | TNBC subtypes | GEPIA2 expression  Basal-like / Triple negative (135) |
| --- | --- | --- | --- | --- |
| TRIML2 | High | TRIM2 | BL1/IM/M/MSL/LAR | High* |
|  |  | TRIM22 | IM/M/MSL | Low |
|  |  | TRIM52 | BL1/MSL/LAR | Low |
|  |  | TRIM68 | MSL | Low |
|  |  | TRIM8 | MSL | Low |
|  |  | TRIM24 | MSL | Similar across subtypes |
|  |  | TRIM38 | IM | Similar across subtypes |
|  |  | TRIM14 | IM/M | Similar across subtypes |
|  |  | TRIM16 | BL2 | High |
|  |  | TRIM29 | BL1/M/LAR | High* |
|  |  | TRIM34 | M/LAR | Low |
|  |  | TRIM3 | LAR | Low* |
|  |  | TRIM36 | LAR | Low |
|  |  | TRIM62 | LAR | Low |
| FOSL1 | High* | FOS | BL1/IM/MSL | Low* |
|  |  | FOSB | IM/MSL | Low* |
|  |  | FOSL2 | BL2 | Similar across subtypes |
| IL1A | High | IL1R1 | BL1/MSL/LAR | Low |
|  |  | IL1R2 | BL1/ M/MSL/LAR | High* |
|  |  | IL1RN | M/MSL | Similar across subtypes |
| CSF2 | High | CSF1R | IM/MSL | Similar across subtypes |
|  |  | CSF2RA | IM | Similar across subtypes |
|  |  | CSF2RB | IM/M/MSL | Similar across subtypes |
| SFRP5 | Similar across subtypes | SFRP1 | BL1/BL2/M/LAR | High* |
|  |  | SFRP4 | BL1/MSL/LAR | Low* |
| DUSP7 | High | DUSP1 | BL1/MSL | High |
|  |  | DUSP4 | BL1/M/MSL/LAR | Low* |
|  |  | DUSP5 | M/MSL/LAR | Low* |
|  |  | DUSP6 | BL1/IM/MSL/LAR | Low* |
|  |  | DUSP10 | M/LAR | Low* |
|  |  | DUSP12 | MSL/LAR | Similar across subtypes |
| IFNK | Low | IFNGR1 | BL2/MSL | Similar across subtypes |
|  |  | IFNG | IM | Similar across subtypes |
| WNT7A | Low | WNT5A | BL2/LAR | Low |
| OR10Y1P | Low | - | - | - |
| CDA | Similar across subtypes | - | - | - |
| CALB1 | Similar across subtypes | CALB2 | BL1/IM/LAR | High* |
| PRR9 | Low | PRR4 | BL1 | High* |
|  |  | PRR13 | LAR | Low |
|  |  | PRR5 | LAR | Similar across subtypes |
| DEFB103A | Low | DEFB1 | BL1/BL2/M/MSL/LAR | High* |
| KRTAP11-1 | Low | KRT5 | BL1/BL2/IM/M/MSL | High* |
|  |  | KRT6A | BL2/IM/M/MSL | High* |
|  |  | KRT6B | BL1/BL2/IM/M/MSL | High* |
|  |  | KRT14 | BL1/BL2/IM/M/MSL | High* |
|  |  | KRT15 | BL1/BL2/IM/M/MSL | High* |
|  |  | KRT16 | BL2/IM/M/MSL | High* |
|  |  | KRT17 | BL2/IM/M/MSL | High* |
|  |  | KRT18 | IM | Low* |
|  |  | KRT19 | BL1/BL2/M | Low* |
|  |  | KRT23 | BL1/BL2/IM/M/MSL | High* |
|  |  | KRT81 | BL1/BL2/IM/M/MSL | High* |
| *Comparing across subtypes BRCA, Log_2_FC Cutoff: 1, p-value Cutoff: 0.05, Match TCGA normal and GTEx data. | | | | |


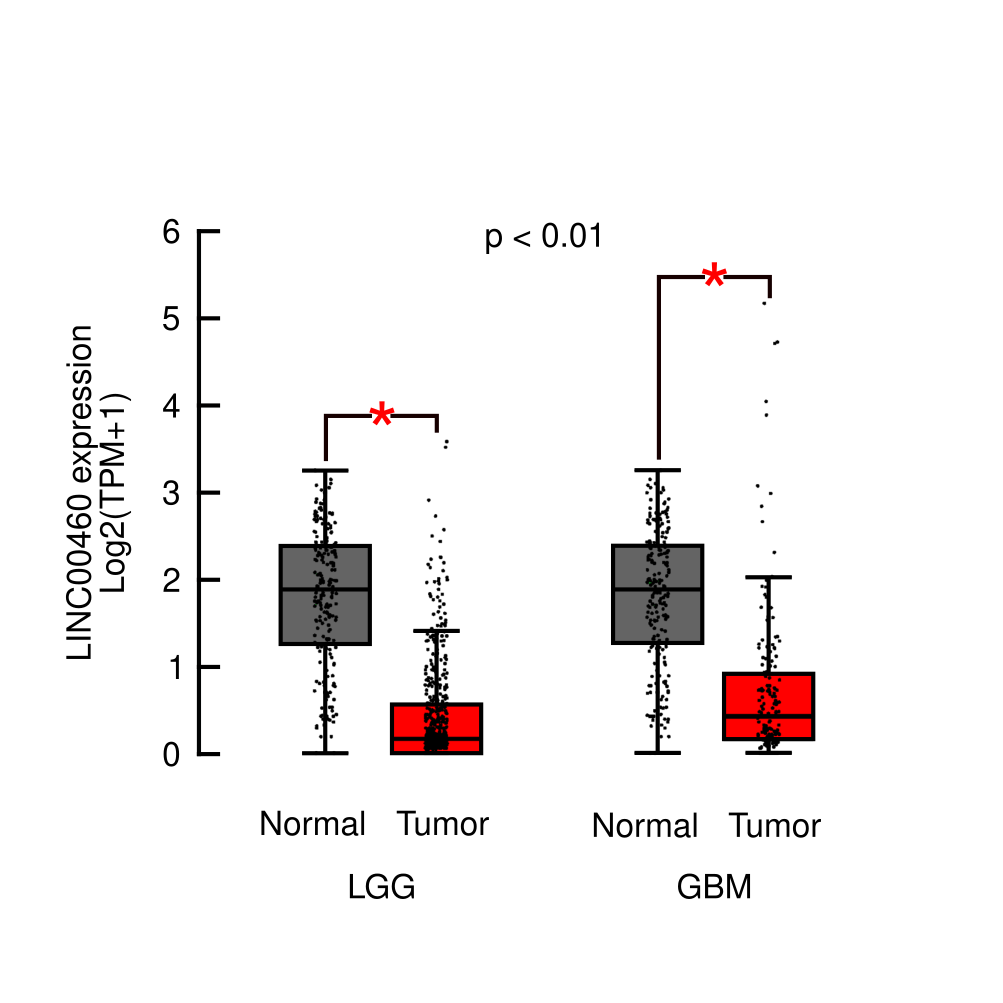


**Figure S1.** LINC00460 over- expression is associated with two novel (not previously reported) central nervous system tumors: GBM and LGG in the TCGA datasets.


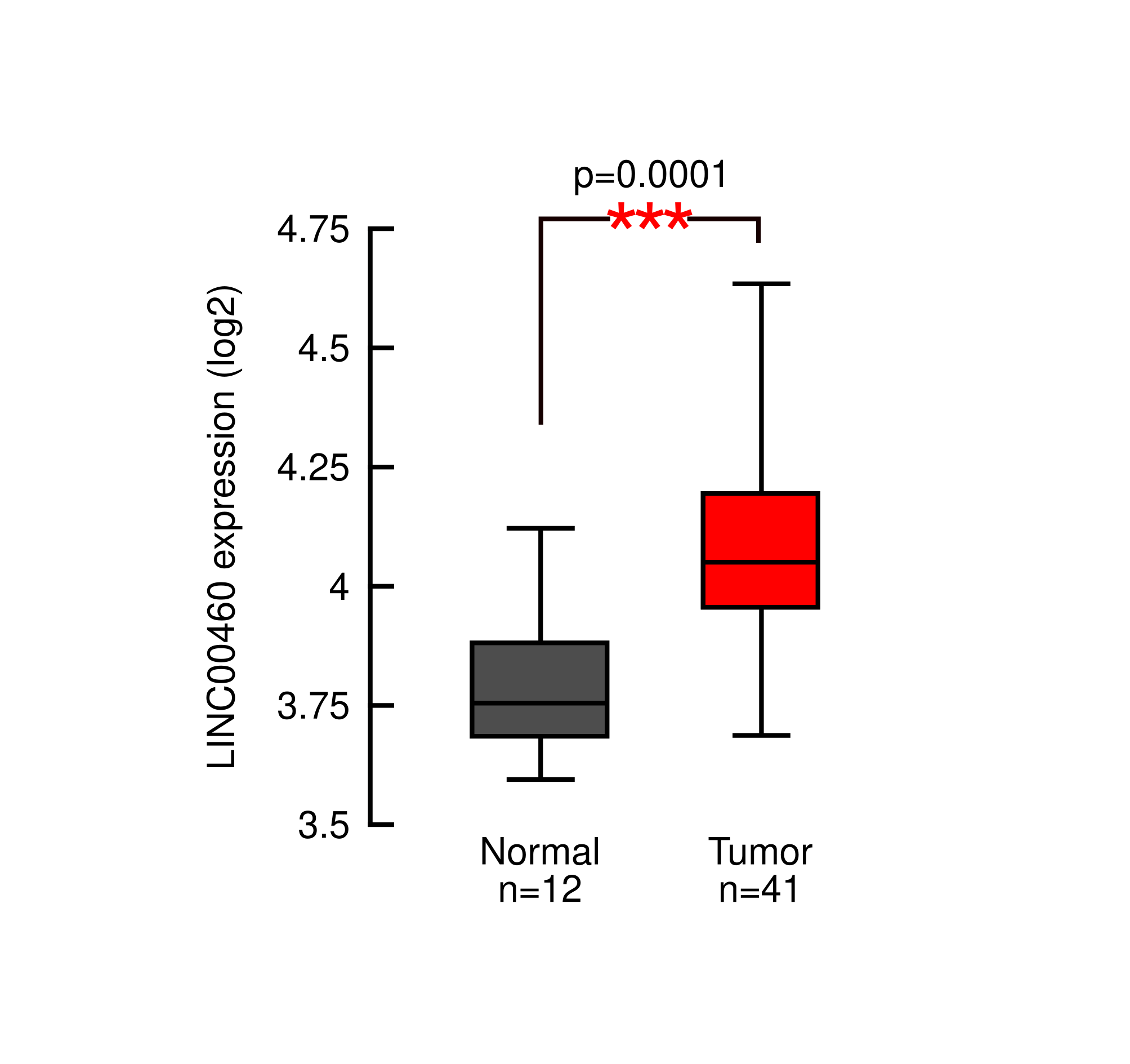


**Figure S2.** LINC00460 high expression is significantly associated with BRCA, compared with normal tissues in the independent GEO cohort GSE29431.


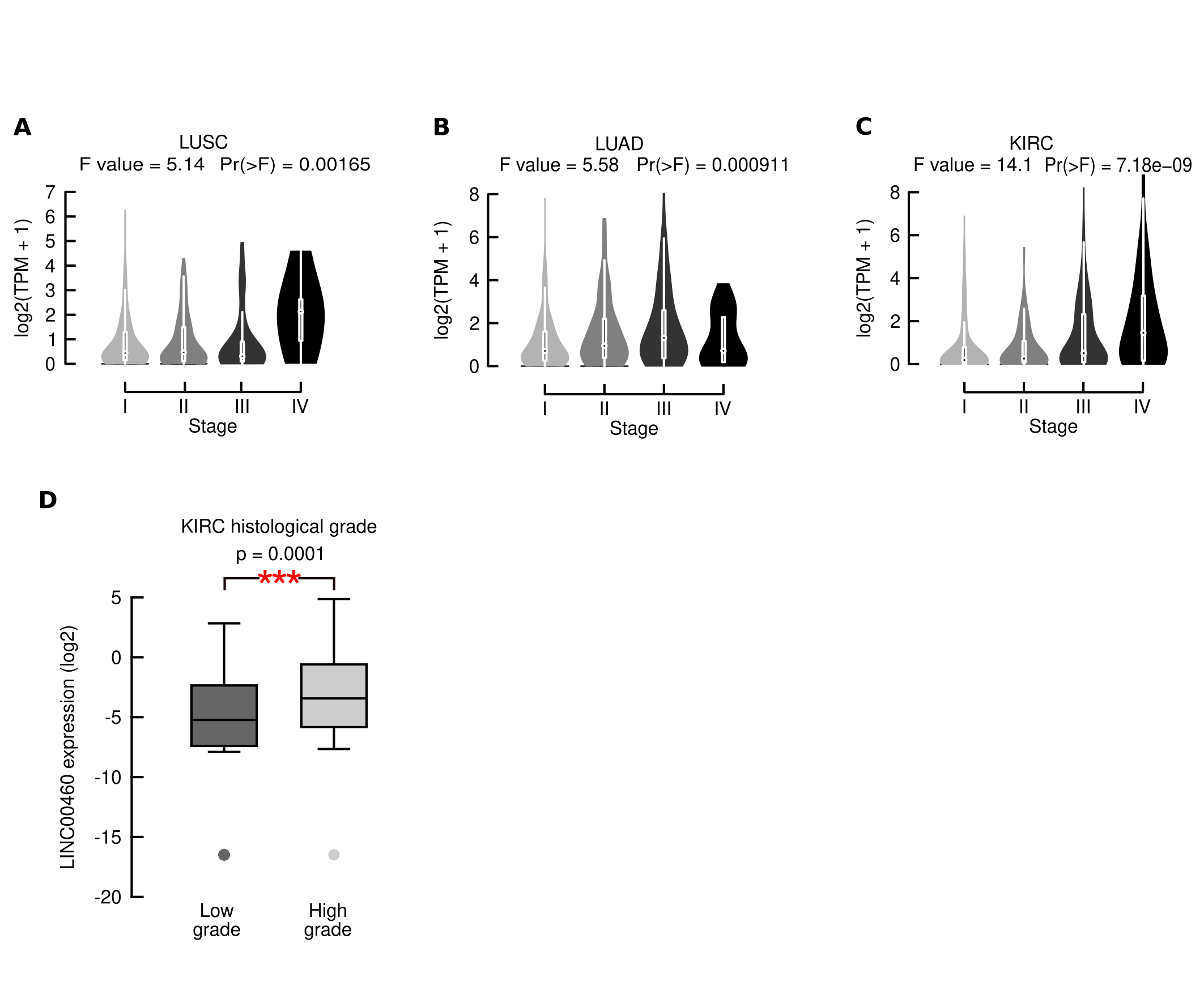


**Figure S3.** LINC00460 high expression is associated with aggressive phenotypes in different TCGA tumors. High expression of LINC00460 is associated with **(A)** advanced stage IV LUSC, **(B)** locally advanced stage III LUAD, (**C)** stage IV KIRC and **(D)** high KIRC histological grade.


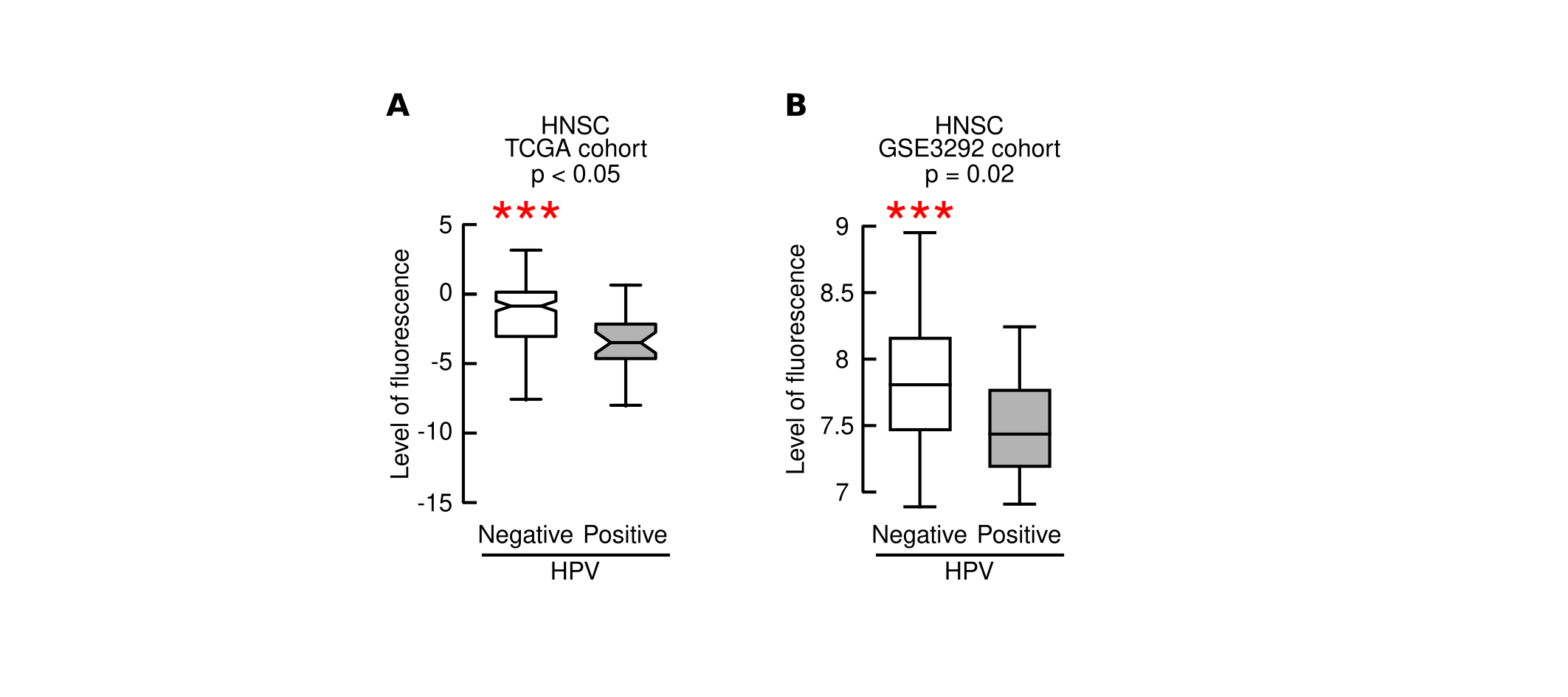


**Figure S4.** LINC00460 over-expression is related to aggressive negative HPV status in two independent HNSC cohorts (A) TCGA cohort, (B) GEO GSE3292 cohort.


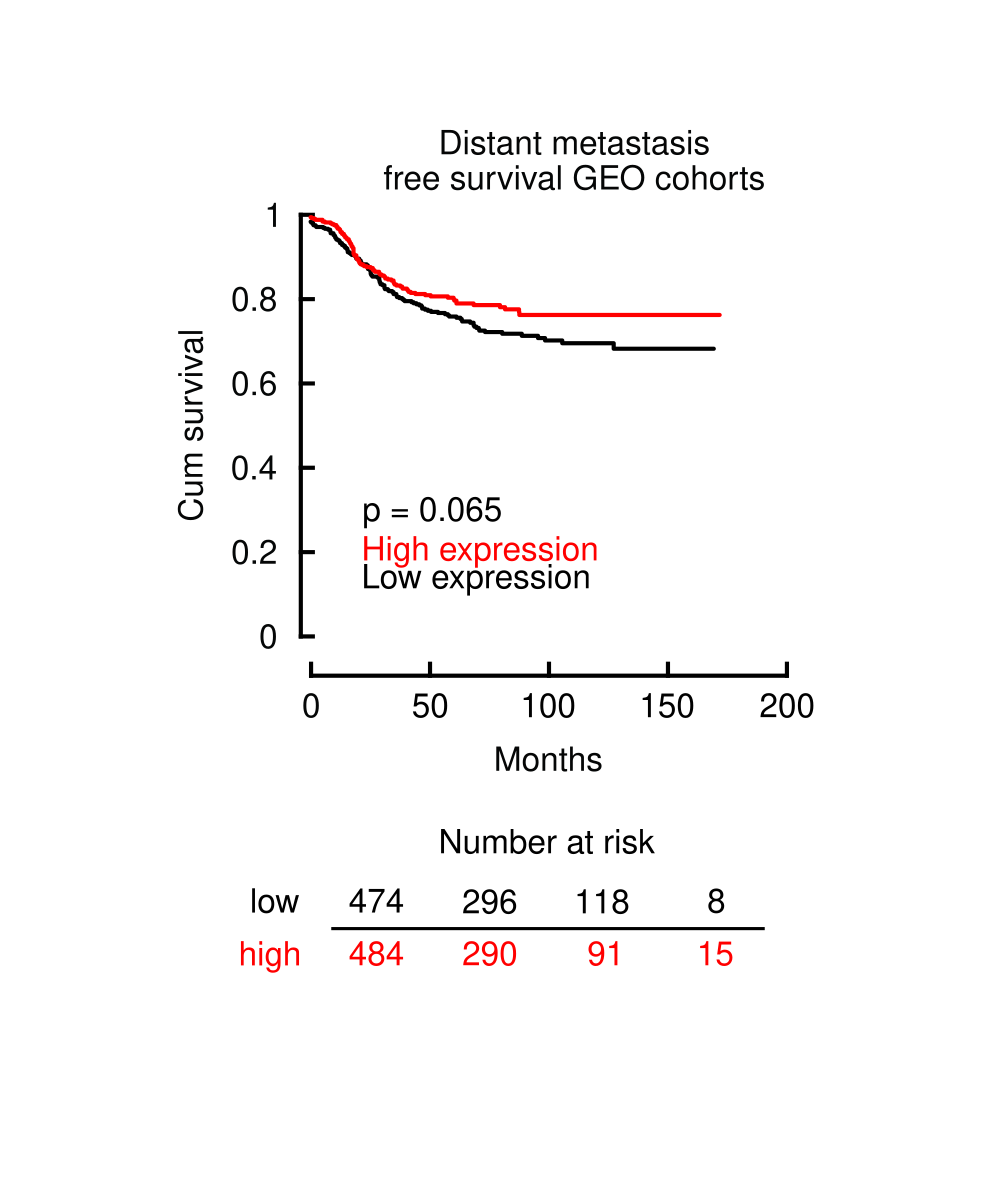


**Figure S5.** Distant metastasis free survival is marginally increased in BRCA patients from the GSE21653 cohort.


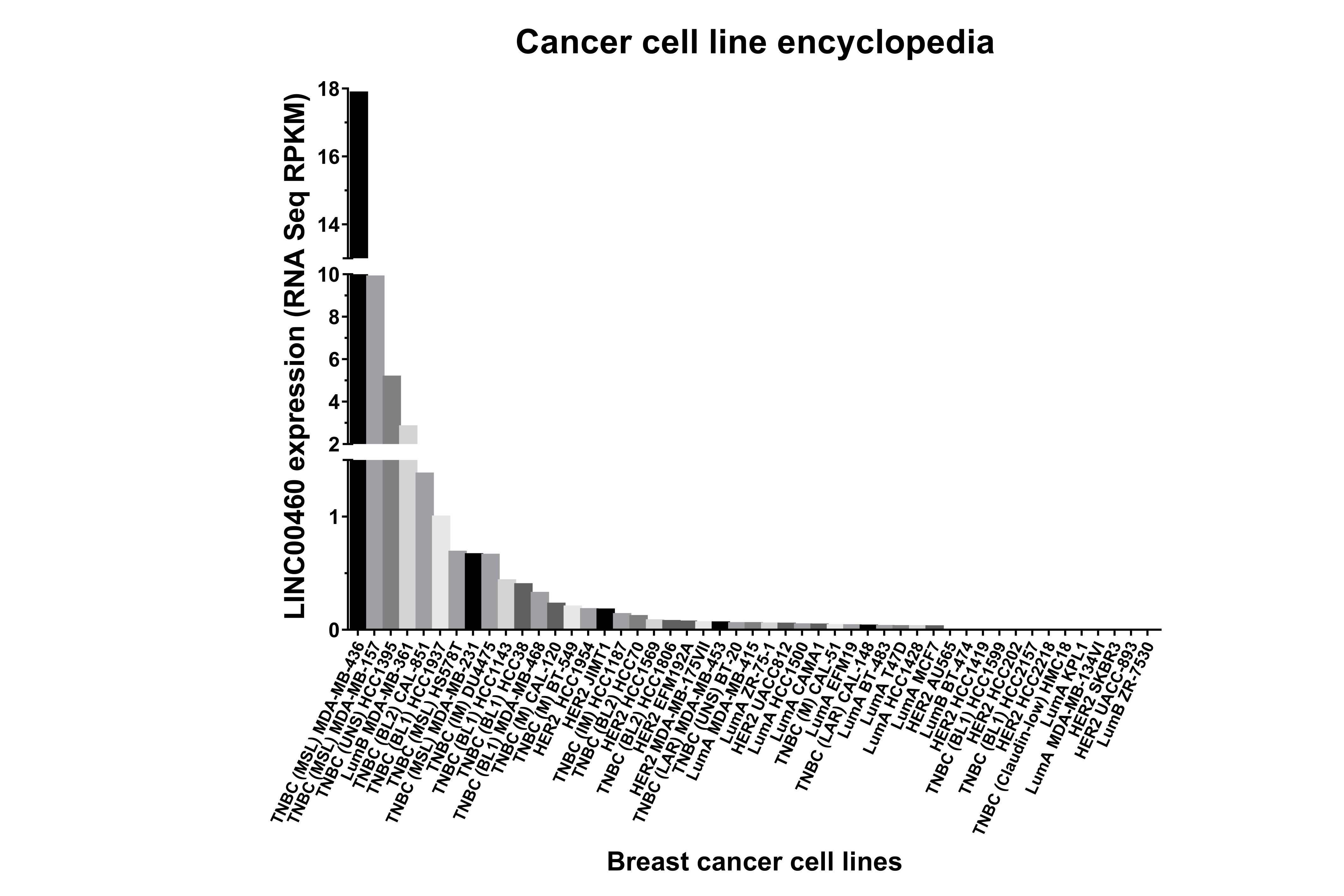


**Figure S6.** LINC00460 expression is enriched in TNBC cell lines in the CCLE.


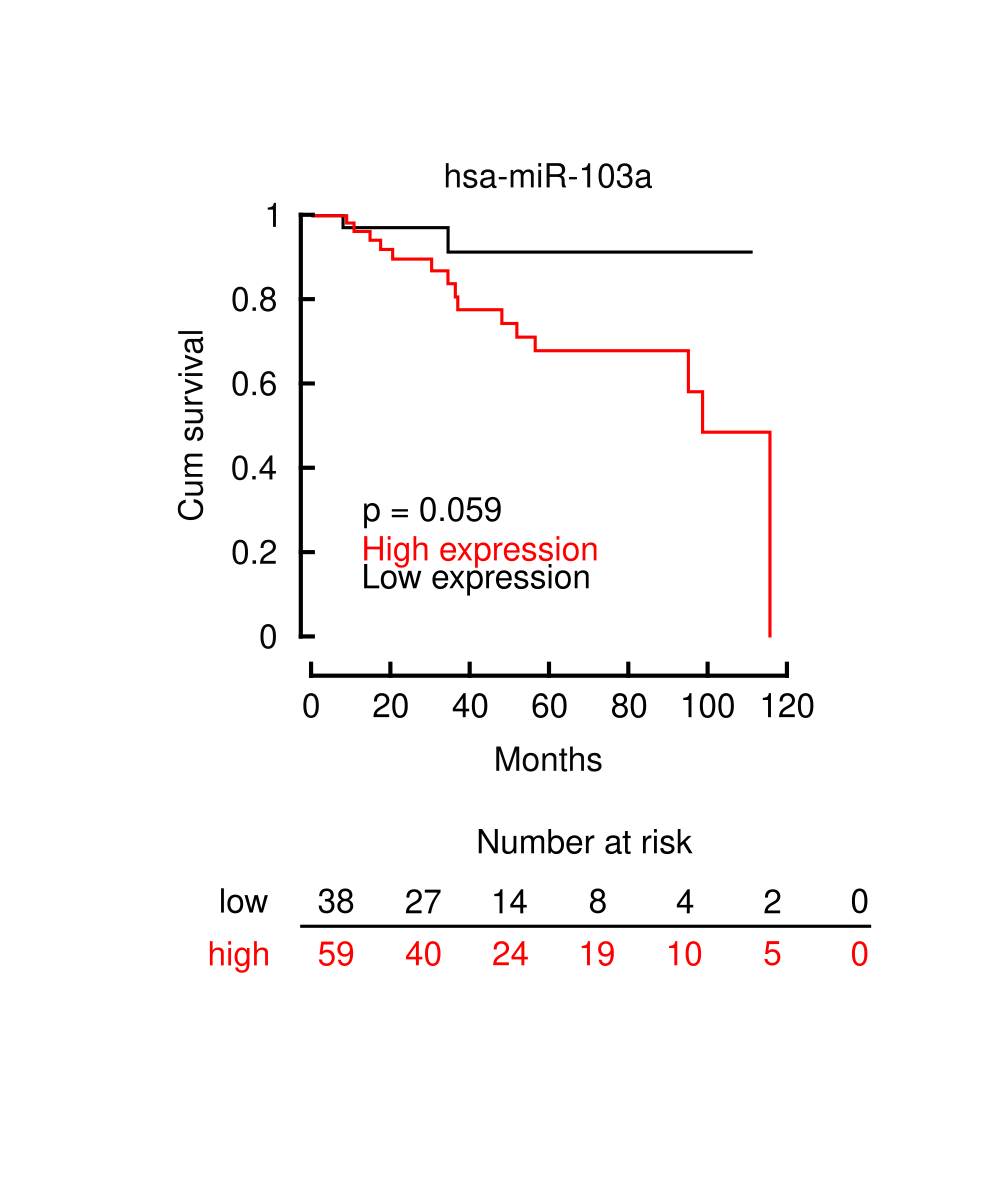


**Figure S7.** The mature sequence of miR-103-a marginally associates with decreased OS in TNBC sing the TCGA cohort.
